# Oral Administration of Bovine and Porcine Milk Exosome Alter miRNAs Profiles in Piglet Serum

**DOI:** 10.1038/s41598-020-63485-8

**Published:** 2020-04-24

**Authors:** Delin Lin, Ting Chen, Meiying Xie, Meng Li, Bin Zeng, Ruiping Sun, Yanling Zhu, Dingze Ye, Jiahan Wu, Jiajie Sun, Qianyun Xi, Qingyan Jiang, Yongliang Zhang

**Affiliations:** 1 National Engineering Research Center For Breeding Swine Industry, Guangdong Provincial Key Laboratory of Agro-Animal Genomics and Molecular Breeding, Guangdong Province Research Center of Woody Forage Engineering and Technology, Guangdong Provincial Key Laboratory of Animal Nutrition Control, South China Agricultural University, 483 Wushan Road, Guangzhou, 510642 China; 2 Institute of Animal Science and Veterinary Medicine, Hainan Academy of Agricultural Sciences, Haikou, 571100 China

**Keywords:** RNA, Transcriptomics

## Abstract

Breast milk is the most important nutrient source for newborn mammals. Studies have reported that milk contains microRNAs (miRNAs), which are potential regulatory components. Currently, existing functional and nutritional two competing hypotheses in milk field though little date have been provided for nutritional hypothesis. In this study, we used the qRT-PCR method to evaluated whether milk miRNAs can be absorbed by newborn piglets by feeding them porcine or bovine milk. The result showed that miRNA levels (miR-2284×, 2291, 7134, 1343, 500, 223) were significantly different between bovine and porcine milk. Four miRNAs (miR-2284×, 2291, 7134, 1343) were significantly different in piglet serum after feeding porcine or bovine milk. After separated milk exosomes by ultracentrifugation, the results showed the selected milk miRNAs (miR-2284×, 2291, 7134, 1343) were present in both exosomes and supernatants, and the miRNAs showed the coincidental expression in IPEC-J2 cells. All our founding suggested that the milk miRNAs can be absorbed both *in vivo* and *in vitro*, which will building the foundation for understanding whether these sort of miRNAs exert physiological functions after being absorbed and provided additional evidence for the nutritional hypotheses.

## Introduction

Breast milk is the first and most important source of nutrition for newborn mammals^1^. By differential centrifugation, milk can be divided into milk fat, whey, casein, cells, and debris and further separated by ultra-centrifugation into extracellular vesicles (EVs) and supernatant^2^.

Exosomes, which are EVs of 30–100 nm in diameter and of endocytic origin, are released by numerous cells and are present in several body fluids, including saliva^3,4^, plasma^5^, urine^6^, amniotic fluid^7^, malignant ascites^8^, bronchoalveolar lavage fluid^9^, and synovial fluids^10^. Studies have reported that exosomes contain lipids, proteins, mRNA, and microRNA (miRNA)^11–14^ and that they serve as novel vehicles in cell-to-cell communication^15,16^. Just like other body fluids, milk contains EVs^2,17^. Hata *et al*. detected the presence of mRNA and miRNA in bovine milk-derived vesicles^18^.

MiRNAs represent a class of endogenous non-coding RNAs of approximately 22 nucleotides in length that are widely distributed in eukaryotes. The biological function of miRNAs is to destabilize mRNAs or halt mRNA translation^19,20^. Studies have reported that 12 body fluids contain miRNAs, and milk has the highest concentration of total RNA that is rich in miRNAs^21^. Milk components that contain miRNAs include milk fat globules^22^, whey^23,24^, and exosomes^11,15^. Interestingly, Izumi *et al*. suggested that miRNAs were also present in the supernatant of ultra-centrifuged bovine raw milk^25^. Furthermore, Zhou *et al*. confirmed the presence of 452 pre-miRNAs in human milk exosomes, which lead to 602 mature miRNAs^26^. Chen *et al*. reported the presence of 245 miRNAs in bovine milk^27^, and Kosaka *et al*. detected 281 of 723 known human miRNAs in human milk by microarray technology^28^. Porcine milk exosomes contain more than 180 pre-miRNAs^29^, and 491 miRNAs have been detected in porcine exosomes by Solexa sequencing^30^. Title *et al*. concluded that up to 635 miRNAs were expressed in a single milk clot sample, with an average of 506 miRNAs per sample^31^.

MiRNAs, which target approximately 60% of genes in mammals^32,33^, are involved in immune function^28,34^, development^35–37^, differentiation^38–40^, proliferation^41–43^ and metabolism^44,45^. MiRNAs play important roles in the regulation of immune cell development, innate immune responses, and acquired immune responses^46,47^. Our previous findings revealed that porcine milk exosomes promote IPEC-J2 proliferation^48^. Even though milk exosomes increase the stability of miRNAs, it is not known whether miRNAs can be absorbed through the digestive tract. Wolf *et al*. reported that miRNAs in bovine milk are transferred among animal species by dietary means because bovine milk exosomes can be absorbed by human and rat intestinal cells^49^. Kosaka *et al*. suggested that breast milk miRNAs could be transferred from mother to infant through dietary intake^28^. However, some published gave the opposing viewpoint, they performance the experiments on two transgenic models, miRNA knock-out and over-expressing mice, which those models may be inappropriate to study the physiological transfer the miRNAs to the newborns of their aberrant miRNA expression, thus, the results showed there were no evidence of miRNA absorption^31,50^. More importantly, there is no information on miRNA absorption in pigs, which are similar to humans in body size. To evaluate whether milk-derived miRNA is absorbed in newborn piglets, we used bovine and porcine milk, which have different miRNA expression profiles, for *in vivo* and *in vitro* experiments. This study will provide evidence on miRNA absorption in newborn mammals.

## Results

### MiRNA comparisons between bovine and porcine milk

**Figure 1:**
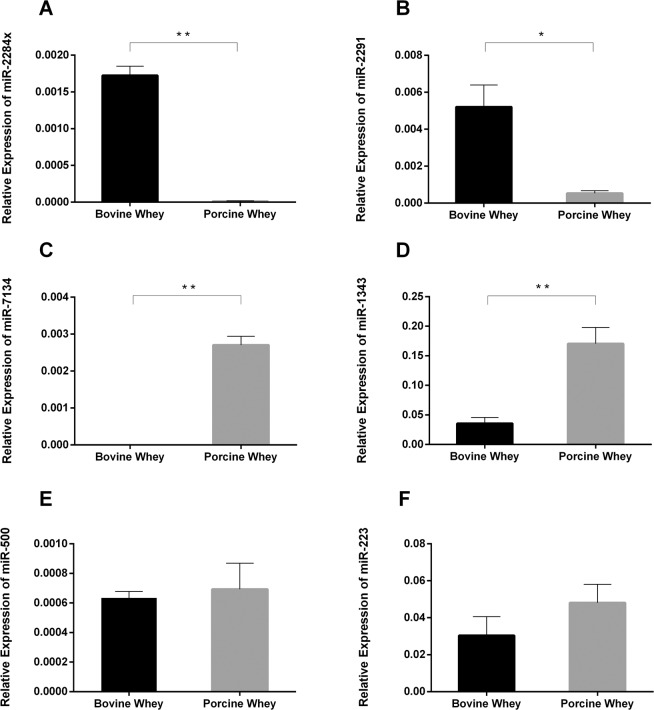
Expression of miRNAs in bovine and porcine whey. Levels of miR-2284×(**A**), miR-2291 (**B**), miR-7134 (**C**), miR-1343 (**D**), miR-500 (**E**), and miR-223 (**F**) in bovine and porcine whey. The data were analyzed by t-test with n = 7 biological replicates. The graph was generated using GraphPad Prism 6. *p < 0.05, **p < 0.01.

Using bioinformatical comparisons with reported bovine^18,27,51^ and porcine^29,30^ milk miRNAs, we selected six miRNAs for further validation: miR-1343, miR-223, miR-2284×, miR-2291, miR-500, and miR-7134. The results of qRT-PCR revealed that miR-2284× and miR-2291 were significantly higher in bovine whey than in porcine whey (Fig. 1A–B). In contrast, miR-7134 and miR-1343 were significantly higher in porcine whey than in bovine whey (Fig. 1C–D). There were no differences in miR-500 and miR-223 levels between bovine and porcine whey samples (Fig. 1E–F).

### MiRNAs in piglet serum after feeding porcine or bovine milk

**Figure 2:**
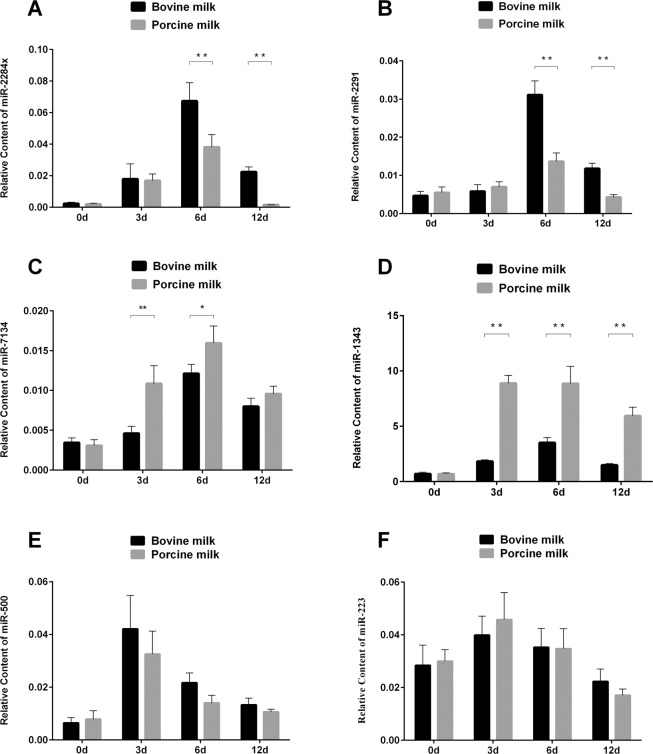
Levels of miRNAs in piglet serum after feeding bovine or porcine milk. Relative levels of serum miRNA, miR-2284×(**A**), miR-2291 (**B**), miR-7134 (**C**), miR-1343 (**D**), miR-500 (**E**), miR-223 (**F**) on days 0, 3, 6, and 12 post-birth. The data were analyzed by ANOVA with n = 9 biological replicates. The graph was generated using GraphPad Prism 6. *p < 0.05, **p < 0.01.

To assess whether milk-derived miRNAs can be absorbed by neonates, we measured the levels of miR-2284×, miR-2291, miR-7134, miR-1343, miR-500, and miR-223 in piglet serum after feeding bovine or porcine milk on four time points (day 0, 3, 6, 12). The results showed that miR-2284×and miR-2291 level were remarkably higher in the bovine milk-feeding group than in the porcine milk-feeding group on day 6 and day 12 and no difference expression on day 0 and 3 (Fig. 2A-B). In contrast, miR-7134 was significantly higher in the porcine milk-feeding group than in the bovine milk-feeding group on days 3 and 6 (Fig. 2C), and miR-1343 was significantly higher in the porcine milk-feeding group at all experimental time points except on day 0 (Fig. 2D). There were no significant differences in the levels of miR-500 and miR-223 between the two groups (Fig. 2E–F). Interestingly, these results were coincidental with the corresponding miRNA levels in bovine and porcine milk whey (Fig. 1). These results indicated that milk-derived miRNAs can be absorbed by newborn piglets and exhibited different content profiles among days, that maybe relevant to diverse physiological requirement after birth.

### MiRNA target prediction

Table 1 shows the predicted target genes for selected miRNAs. MiR-1343 attenuates porcine adipose triglyceride lipase (*ATGL*) and TGF-β receptors^52^. Owing to miR-2284×, miR-2291, miR-7134, and miR-1343 had dramatically differences between two kinds of whey and feeding experiments, we selected these four miRNAs for *in vitro* absorption experiments.

**Table 1:** Target genes of selected miRNAs.

Gene name	Target mRNA	NCBI Reference Sequence	Score	Energy (kCal/Mol)
miR-2291	SIX homeobox 4 (*SIX4*)	NM_001244614	153	−22.89
	Proline rich and Gla domain 4 (*PRRG4*)	NM_001244836	158	−21.09
	StAR related lipid transfer domain containing 4 (*STARD4*)	NM_001143726	157	−21.6
	Mannosyl (alpha-1,3-)-glycoprotein beta-1,2-N-acetylglucosaminyltransferase (*MGAT1*)	NM_001078668	153	−22.45
	Phosphatidylinositol-4-phosphate 5-kinase type 1 alpha (*PIP5K1A*)	NM_001244451	160	−22.26
	Capping actin protein of muscle Z-line beta subunit (*CAPZB*)	NM_001113444	167	−21.83
	Matrix metallopeptidase 14 (membrane-inserted) (*MMP14*)	NM_214239	159	−23.71
	Synaptojanin 2 binding protein (*SYNJ2BP*)	NM_001244991	162	−24.44
	Beta-secretase 1 (*BACE1*)	NM_001289854	164	−20.33
	Peroxiredoxin 6 (*PRDX6*)	NM_214408	154	−21.74
	Surfactant protein A1 (*SFTPA1*)	NM_214265	150	−20.49
	Thymidylate synthetase (*TYMS*)	NM_001243579	158	−20.55
	CCCTC-binding factor (zinc finger protein) (*CTCF*)	NM_001244660	156	−23.04
miR-7134	Intercellular adhesion molecule 3 (*ICAM3*)	NM_001145379	167	−22.74
	Cell death inducing p53 target 1 (*CDIP1*)	NM_001244099	159	−20.63
	Solute carrier family 7 (cationic amino acid transporter, y+ system), member 1 (*SLC7A1*)	NM_001012613	153	−20.01
	Cornichon homolog (Drosophila) (*CNIH*)	NM_001243525	154	−20.62
miR-2284×	Versican (VCAN)	NM_001206429	154	−22.94
	Pygopus family PHD finger 2 (*PYGO2*)	NM_001185175	153	−20.34
	SLIT and NTRK like family member 1 (*SLITRK1*)	NM_001308829	165	−24.86

### MiRNAs in milk-derived exosomes and exosome-free whey

**Figure 3:**
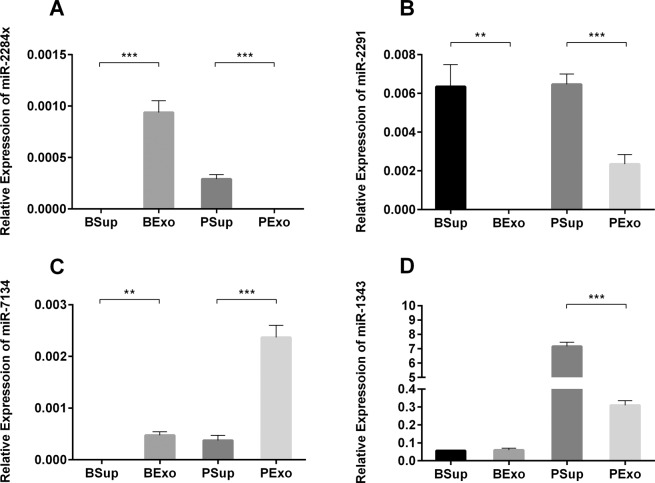
Levels of miRNAs in exosomes and supernatants of bovine and porcine milk. Relative levels of miR-2284×(**A**), miR-2291 (**B**), miR-7134 (**C**), and miR-1343 (**D**). Abbreviations: BSup, bovine milk supernatants; BExo: bovine milk exosomes; PSup: porcine milk supernatants; PExo: porcine milk exosomes. The data were analyzed by t-test with n = 8 biological replicates. The graph was generated using GraphPad Prism 6. **p < 0.01, ***p < 0.001.

To measure the levels of exosomal and non-exosomal miRNAs in bovine and porcine milk, we collected milk-derived exosomes and supernatants for western blot (Supplementary Figure) and qRT-PCR detection. The results revealed that miR-2284× was significantly higher in bovine milk exosomes than in bovine milk supernatant. Opposite results were obtained with porcine milk (Fig. 3A). MiR-2291 was higher in supernatants than in exosomes of both bovine and porcine milk samples (Fig. 3B). In contrast, miR-7134 was significantly higher in exosomes than in supernatants of both bovine and porcine whey (Fig. 3C). MiR-1343 was significantly higher in supernatants than in exosomes of porcine whey, however, there were no differences in bovine milk (Fig. 3D). These results revealed that milk-derived miRNAs are present in different forms and that the distribution of miRNAs in milk may differ among species.

### Milk-derived exosomes and exosome-free whey affected the concentration of corresponding miRNAs in IPEC-J2 cells

**Figure 4:**
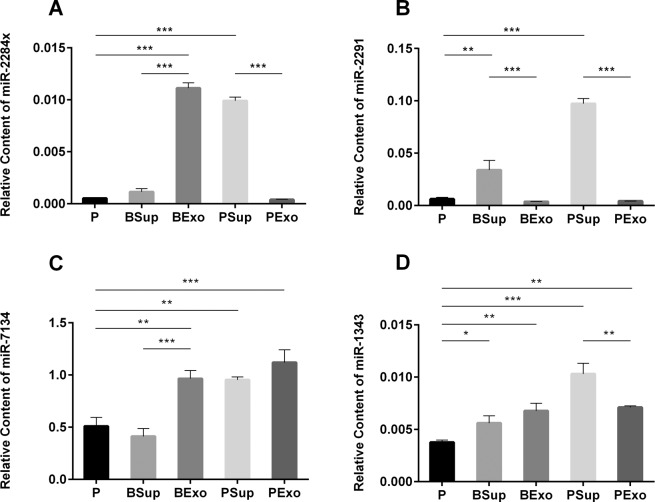
Levels of miRNAs in IPEC-J2 cells following incubation with bovine/porcine milk exosomes and supernatants. MiR-2284× levels increased following treatment with BExo and PSup (**A**), and miR-2291 levels were significantly higher in both BSup and PSup (**B**). All treatments significantly increased miR-7134 levels except for BSup (**C**) and significantly increased miR-1343 levels (**D**). Abbreviations: P: PBS; BSup: bovine milk supernatants; BExo: bovine milk exosomes; PSup: porcine milk supernatants; PExo: porcine milk exosomes. The data were analyzed by ANOVA with n = 6 biological replicates. The graph was generated using GraphPad Prism 6. *p < 0.05, **p < 0.01, ***p < 0.001.

To evaluate whether exosomal and non-exosomal miRNAs are absorbed by IPEC-J2 cells, we measured the relative levels of miR-2284×, miR-2291, miR-7134, and miR-1343 in cells following incubation with bovine and porcine milk exosomes and supernatants. The results revealed that incubation with bovine/porcine milk exosomes and supernatants increased the levels of miRNAs in IPEC-J2 cells (Fig. 4A–D), and U6 among groups had consistent level (data not shown). Higher miRNA levels in the samples resulted in higher miRNA levels in IPEC-J2 cells. Therefore, both exosomal and non-exosomal miRNAs can be absorbed by IPEC-J2 cells.

## Discussion

Breast milk not only the primary source of nutrition for newborn mammals but also can as a potential immunoprotector and developmental regulators for infant and mother^53^, epigenetic regulators^54,55^, metabolism regulators^56^, disease biomarkers^57^ and so on. Studies have reported that mammalian milk, such as human^26,28^, bovine^18,27,51^, porcine^29,30^, murine^23,31^, and tammar wallaby^58^, contains miRNAs. However, whether milk miRNAs exert any physiological regulation in newborns has not been elucidated. Milk from different species may have different miRNA profiles^25,26,29,30^. Our study findings revealed that the levels of four miRNAs were different between porcine and bovine milk.

MiRNA is degraded by RNase. Exosomes, one of major forms of membrane-bound vesicles, are present in several body fluids^21^. A large proportion of miRNAs are encapsulated in exosomes, and exosomal miRNAs have been detected in different types of mammalian milk through sequencing or microarray technology^25–27,29,30^. As nanoparticles, exosomes confer protection to miRNAs under the harsh extracellular environment of the digestive tract^18,28,51,59^. However, a considerable fraction of milk-derived miRNAs is located in the supernatants. Izumi *et al*. reported that miRNAs in bovine milk were present in both ultra-centrifuged supernatants and exosomes^25^. In this study, we tested the levels of four miRNAs in exosomes and supernatants. In bovine milk, miR-2284x and miR-7134 were present in exosomes, while miR-2291 was present in supernatants. In porcine milk, miR-2284×, miR-2291, and miR-1343 were mostly present in supernatants, while miR-7134 was present in exosomes. Interestingly, other studies found that non-exosomal miRNAs co-fractionated with protein complexes were resistant against degradation. Even though a minority of specific miRNAs is associated predominantly with microvesicles, the majority of miRNAs are bound to Argonaute2 protein in plasma^60,61^. In addition, nucleophosmin 1^62^ and high-density lipoprotein^63^ are two miRNA-binding proteins that play roles in miRNA protection, export, and transport. As the majority composition of breast milk are similar to blood, it is reasonable to speculate that milk-derived miRNAs may be bound to proteins.

However, there were limited points existed in our research about the distribution of miRNAs in milk, some previously publications used different milk isolation methods to revealed the different milk part miRNAs expression patters. For instance, Benmoussa *et al*. reported the characterization of milk EV contain the bulk of milk miRNAs (include bta-miR-125b, bta-miR-148a, etc.), sediment at 12,000 g and 35,000 g, and found their distribution pattern was different from that of exosome-enriched proteins, but similar to that of several proteins commonly found in milk fat globule membranes (MFGM), including xanthine dehydrogenase (XDH)^64^. Gerstl *et al*. applied next generation sequencing and q-PCR identified the miRNA expression profile in the skim and fat fraction of human, goat, and bovine milk as well as infant formulas and found that most of known advantageous miRNAs in exosomes and fat layer were very similarity^65^. Munch *et al*. were used the next-gen deep sequencing revealed the miRNAs profile in the lipid fraction of human breast milk and found that known and novel miRNAs were enriched in breast milk fat globules, and expression of several novel miRNA species were regulated by maternal diet^66^. From above researches we can know that different parts of milk would be contain similar miRNAs species and which would be change the expression the miRNA expressions in the infants after feeding mammals or incubated with other cells, and in our research we only considered the miRNAs in supernatant and exosome part of bovine and porcine milk (Fig. 3 and Fig. 4) for their forms, distribution and absorb ability, which would be need for further experimental research to identify the exactly distribution of those miRNAs in milk part and their transfer approach or functions.

Additionally, the separation method of milk-derived extracellular vesicles is important for RNA enriched and would lead to different biological functions of the EVs. Gerstl *et al*., obtained skim milk (6,500 g, 30 min, 4 °C to 12,000 g, 1 h, 4 °C) or fat layer (6,500 g, 30 min, 4 °C) by different centrifuged speed, collecting milk exosome by ExoQuick kit shown the miR-148a is highly conserved in human, bovine and goat milk^65^. Rubio *et al*. identified miRNAs, piRNAs, tRNAs, snRNAs, and snoRNAs in milk/plasma centrifugation at 16,000 g for 15 minutes at 4 °C^55^. Herwijnen *et al*., collected sucrose gradient (1.12–1.18 g/ml) fractions from human and porcine milk showed abundant of let-7 family members and miR-148a^67^, a series of centrifugations and filtrations combination of ExoQuick regent for human milk exosome isolation was proofed that miRNA-148a is a highly expressed miRNA and down-regulated PTEN (phosphatase and tensin homolog) in normal fetal colon epithelial but not in colon tumor cells, and milk-derived exosomes deleted of miRNA-148a, which inhibited proliferation and DNMT1(DNA methyltransferase 1) expression in cells^68^. But a recently reported that unfractionated cow milk and derived EV subsets with differential ultracentrifugation 12,000 g (P12K), 35,000 g (P35K), 70,000 g (P70K), and 100,000 g (P100K) exhibited P100K EV were enriched in reference miRNA sequences, and P12K and P35K EV in related isomiR. Milk EV miR-223 was transferred in cells and down-regulated the reporter gene^69^. All those evidence in hinted the separation methods of milk EV will not only affect the non-miRNA’ concentration and form enriched but also their bioactivity. In our research, the ultracentrifugation was used for exosome and exosome-free separation showed the miRNAs (miR-2284×, miR-2291, miR-7134, miR-1343) absorbability coincide within pig serum and cells suggested those separation conditions facility the specific miRNAs gained in milk and stabilize for their absorption or function’s regulation, but need for further identification.

To investigate whether milk-derived miRNAs are absorbed, we designed an *in vivo* experiment using piglets and an *in vitro* experiment using IPEC-J2 cells. The *in vivo* and *in vitro* results revealed that milk miRNAs were absorbed by cells in the digestive tract. In addition, miRNAs in exosomes and supernatants were absorbed by IPEC-J2 cells, and the levels of miRNAs in cells were in agreement with the levels of miRNAs in milk (Fig. 3). There is conflicting information on the absorption of milk-derived miRNAs. Baier *et al*. reported that milk-borne miRNAs can be absorbed by humans^59^, and Chen *et al*. reported that miRNAs in milk exosomes can be absorbed by IPEC-J2 cells^48^. Furthermore, Sun *et al*. demonstrated that colostrum’s MVs may transfer immune-related miRNAs into cells and exert immunomodulatory effects^34^. Izumi *et al*. revealed that bovine milk exosomes containing RNA may enter human macrophages^25^. Gerstl *et al*. identified that the high expression miR-148a-3p in milk exosome and fat layer with Exo‐Red labeled can be take into CRL 1831 cells (human normal intestine cell line), K562 (leukemia cells) and Lim 1215 (colon cancer cells) and showed up-regulated in the entered cells^65^. MiRNAs transferred from maternal milk to neonates via the digestive tract are essential to the development of the immune system^26,29^. However, Title *et al*. reported that there is no evidence on milk miRNA absorption in miR-375-knockout and miR-200c/141-knockout mouse models and that miRNAs may be degraded by the digestive system^31^, because those two miRNAs related to control of the exosome endocytosis or exocytosis would influence their uptake, the KO mice were inappropriate models to study milk exosome uptake^70^ and they can’t inferred the milk miRNAs only provide nutrition for offspring. Laubier *et al*. demonstrated that milk-rich miR-30b could not be detected in transgenic pups compared to wild-type pups^50^. Above two studies propounded that milk-derived miRNA cannot be uptaken by pups. Coincidentally, the authors employed gene-changed mice models, which made the context complicated and the whole process biologically artifactual. Otherwise, Manca. *et al*. gained the unique distribution profiles and accumulated in intestinal mucosa, spleen, liver, heart or brain after administered mice with transfected fluorophore-labeled microRNAs into bovine milk exosomes, which provided the experimental evidence for the uptake of miRNAs by newborn^71^. In this study, we utilized wild-type models and provided indirect evidence on the absorption of milk-derived miRNAs.

Based on our *in vivo* and *in vitro* results, breast milk miRNAs can be absorbed through the neonatal digestive tract. As key post-transcriptional gene regulators, miRNAs play important roles in several physiological and pathological processes^72^. Which would be relate to the epigenetic regulatory effect in infants, such as during in the different lactation times different genes will participate the milk fat synthesis and secretion^73^, and the miRNAs could control the homeostatic regulation of cholesterol and triacylglycerol metabolism^74–76^. For instance, the miR-148a showed an higher expression than other lactation-related miRNAs during the lactation mammary glands of the Chinese swamp buffalo^77^, and the miRNA-148a and miRNA-17–5p shown to synergistically increase milk triacylglycerol synthesis via regulation of PPARGC1A and PPARA in goat MECs (mammary epithelial cells)^78^. All those evidence implicated the regulated function of genes or miRNAs would be suit to the infants’ requirements, and we speculated the results of different miRNAs expressions showed time variation in serum after feeding different species milk in our research would be coincide with the pattern.

## Conclusions

In this study, we found that the different miRNAs (miR-2284×, miR-2291, miR-7134 and miR-1343) expression between bovine whey and porcine whey have diverse content profiles in newborn piglets’ serum from two milk-feeding groups. Furthermore, different distribution of miRNAs in porcine and bovine milk format (exosome and exosome-free supernatants) showed the uniform expression pattern in IPEC-J2 cells. These findings contribute to the debate concerning whether milk-source miRNAs can be absorbed by infants, and to building the foundation for understanding whether these sort of miRNAs exert physiological functions after being absorbed.

## Materials and Methods

### Milk samples

Porcine milk samples were collected from healthy lactating Large White pigs one day following parturition. The pigs were bred at the breeding farm of the Livestock Research Institute (Guangzhou, China). Bovine milk samples were collected from healthy one- to five-day old lactating Holstein cows after parturition. The cows were bred at the breeding farm of Feng Xing Milk Company (Guangzhou, China). All milk samples were stored at −80 °C immediately after collection.

### Experimental feedings and serum collection

Three Large White pigs, which were in first parturition and deliveries on the same day, were used in this study. Six newborn piglets from each litter were randomly selected and assigned to one of two groups, a porcine milk-feeding group and a bovine milk-feeding group, with nine piglets per group. The porcine milk-feeding group received milk from the sow, while the bovine milk-feeding group received bovine milk artificially. Blood samples (5 mL) were collected from the anterior vein of the piglets on day 0, 3, 6, and 12 after birth. The serum was separated by centrifugation and stored at −80 °C.

### Whey preparation

Porcine and bovine milk samples were centrifuged twice at 1,200 × g for 10 min at 4 °C to remove milk fat and mammary gland-derived cells. Defatted milk samples were centrifuged at 20,350 × g for 60 min at 4 °C to remove residual fat, casein, and other debris (modified from Izumi *et al*.^51^). The clear supernatant (whey) was collected for further use.

### Preparation of exosome and exosome-free supernatants

The collected whey was further ultra-centrifuged at 110,000 × g for 2 h at 4 °C in an SW41T rotor (Beckman Coulter Instruments, Fullerton, CA) to precipitate the exosomes^30^. After ultra-centrifuged we collected the pellet as the milk exosome in under layer and exosome-free supernatants in upper layer of the centrifuge tube. The pellet was washed with PBS and ultra-centrifuged to purify the exosomes while the exosome-free supernatants were carefully stored at –80 °C directly. Finally, the purified exosomes were re-suspended in 30 mL PBS and stored at −80 °C for used.

### Cell culture

Porcine small intestinal epithelial (IPEC-J2) cells were cultured at 37 °C and 5% CO_2_ in Dulbecco’s modified eagle medium/Ham’s F-12 in a 1:1 ratio (Invitrogen, Life Technologies, Carlsbad, CA, USA) supplemented with 5% fetal calf serum (FCS; Invitrogen) and 5 ng/mL epidermal growth factor (EGF; Peprotech, Rocky Hill, NJ, USA). IPEC-J2 cells were seeded at 0.5 × 10^5^ cells/mL in a 10 mL volume in plastic tissue culture flasks (75 cm^2^ Corning, Corning, NY, USA). After reaching confluency (four days)^79^, the cells were seeded into 6-well tissue culture plates (9.6 cm^2^/well) at 2.0–2.3 × 10^5^ cells/well in a 2 mL volume. The cells were allowed to adhere for 24 h, and the media were replaced every other day. When the cells were 90% confluent, we added 0.5 mL exosomes or exosome-free supernatants to each well, the equal volume PBS added as control. We determined that a 25% media substitution was optimum. Exosomes suspended in PBS and supernatants were passed through 0.45-μm and 0.22-μm membrane filters prior to incubation. IPEC-J2 cells were harvested after 8 h. we give all the *in vitro* experiment for three repeats test.

### RNA extraction and qRT-PCR

Total RNA was isolated from samples using Trizol reagent (Invitrogen, Carlsbad, CA). Trizol reagent (1 mL) was added to 300 µL sample (whey/serum) and to each well (cells). Samples were spiked with 50 fmol synthetic cel-miR-39 as an internal control for extraction efficiency (modified from Kroh *et al*.^80^), and U6 was used as an internal control for cell assay. Total RNA was first digested with DNase I (Promega, Madison, WI, USA), and 100 ng of total whey/serum RNA or 2 μg of total cell RNA was reverse transcribed into poly (A) tail-added cDNA using the Mir-X miRNA First Strand Synthesis kit (Takara Bio Company, Dalian, China). The resulting cDNA was diluted 10-fold with nuclease-free H_2_O. The PCR reaction mixture (20 µL) contained 2 μL template cDNA, 10 μL of 2× Taq Plus Master Mix (Vazyme Biotech Co., Nanjing, China)/GoTaq qPCR Master Mix (Promega, Madison, WI), and 0.5 μL 1 mM of each primer. The PCR products were examined on a 3% agarose gel to confirm that a single PCR product was generated. The real-time PCR thermal profile consisted of 95 °C for 2 min, 40 cycles at 95 °C for 15 s, the annealing temperature for 15 s, and 72 °C for 30 s, followed by the melting curve stage. The miRNA forward primer was designed using Primer 5.0 (Table 2).

**Table 2:** Primers for qRT-PCR.

Gene name	Sequence (5 ′ to 3 ′)
miR-2284×	TGAAAAGTTCGTTCGGGTTTT
miR-2291	GCTGATAGTGAGCGACTGGGGCAG
miR-7134	ATGCGGAACCTGCGGATACGG
miR-1343	CTCCTGGGGCCCGCACTCTC
miR-500	ATGCACCTGGGCAAGGATTCT
miR-223	TGTCAGTTTGTCAAATACCCCA

### MiRNA target prediction

To predict miRNA target sites, we analyzed miRNA targets using miRanda v3.3a microRNA target scanning algorithm^81^ with the default parameters and cutoffs (score ≥ 150 and energy ≤−20.0). Sequences of 3’UTRs of porcine were obtained from NCBI (https://www.ncbi.nlm.nih.gov/).

### Statistical analysis

Data were expressed as mean ± standard error of the mean (SEM). Significant differences were assessed by t-test for two-group comparisons and by one-way analysis of variance (ANOVA), least significant difference (LSD) or Duncan test or Tukey analysis *post hoc* test for multiple comparisons using SPSS 19.0. Statistical significance was set at *p* < 0.05.

### Ethical approval

This article does not contain any studies with human participants performed by any of the authors and all the animal procedures were conducted under the protocol (SCAU-AEC-2016-0714, 14 July 2016) approved by Institutional Animal Care and Use Committee (IACUC) of South China Agricultural University.

### Methods statement

All the experimental procedures were conducted under the protocol (SCAU-AEC-2015–0127, 27 January 2015) approved by the Experimental Operations Management Association (EOMA) of South China Agricultural University.

## Supplementary information

Supplementary Figure 1.

## Data Availability

The datasets generated during and/or analysed during the current study are available from the corresponding author on reasonable request.
