# Supplementary material for: Oral Administration of Bovine and Porcine Milk Exosome Alter miRNAs Profiles in Piglet Serum
Source: Sci Rep. 2020 Apr 24;10:6983. doi: 10.1038/s41598-020-63485-8 (PMC7181743; doi:10.1038/s41598-020-63485-8)
Supplement: Supplementary file 1 — Supplementary Figure 1. [file 41598_2020_63485_MOESM1_ESM.pdf]

Oral Administration of Bovine and Porcine Milk Exosome Alter miRNAs Profiles in Piglet Serum

Delin Lin[†,§], Ting Chen[†,§], Meiying Xie[†], Meng Li[†], Bin Zeng[†], Ruiping Sun[#], Yanling Zhu[†], Dingze Ye[†],Jiahan Wu[†], Jiajie Sun[†], Qianyun Xi[†], Qingyan Jiang[†], Yongliang Zhang[*,†]

[†]: National Engineering Research Center For Breeding Swine Industry, Guangdong Provincial Key Laboratory of Agro-Animal Genomics and Molecular Breeding, Guangdong Province Research Center of Woody Forage Engineering and Technology, Guangdong Provincial Key Laboratory of Animal Nutrition Control, South China Agricultural University, 483 Wushan Road, Guangzhou 510642, China.

[#]: Institute of Animal Science and Veterinary Medicine, Hainan Academy of Agricultural Sciences, Haikou 571100, China.

[§] D.L. and T.C. contributed equally to this work.

[*] Address proofs and correspondence to:

Pro. Yongliang Zhang

College of Animal Science

South China Agricultural University

Wushan Avenue, Tianhe District, Guangzhou, 510642

P.R. China.

E-mail address: Zhangyl@scau.edu.cn

Tel: +86 20 85281269. Fax: +86 20 85280740

Supplementary Figure 1

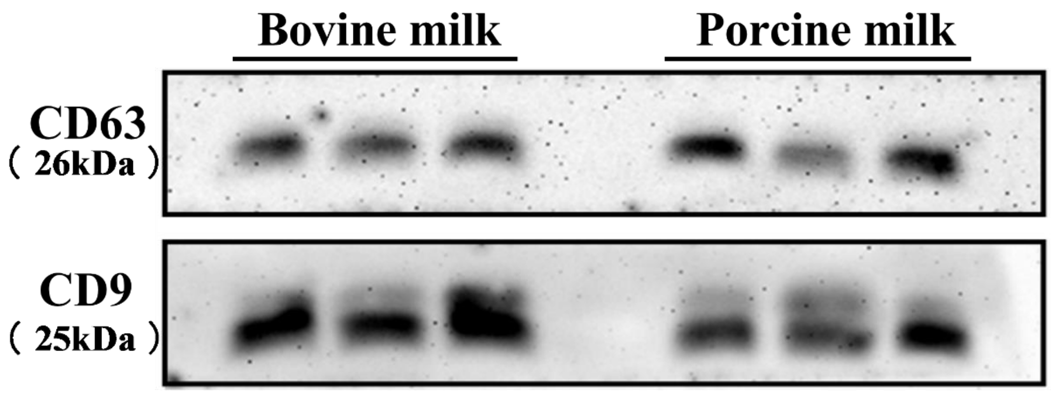

Supplementary Figure1. Identification of exosomal marker proteins in bovine and porcine milk. Exosomal marker proteins CD63 and CD9 were detected from three bovine milk samples and three porcine milk samples by western blotting.
